# Clinical efficacy and prognosis study of recurrent laryngeal nerve anatomical surgery on serum TNF-a, CRP, interleukins IL-6, IL-10, and IL-1b and outcomes

**DOI:** 10.5937/jomb0-56808

**Published:** 2025-08-21

**Authors:** Yongtao Luo, Hui Cheng

**Affiliations:** 1 The Traditional Qilu Medical University, Department of Human Anatomy Teaching and Research Office, Zibo, Shandong Province, China

**Keywords:** TNF-a, CRP interleukins IL-6, IL-10, IL-1b, thyroidectomy, recurrent laryngeal nerve injury, anatomical exposure, vocal cord function, inflammatory markers, interleukins, quality of life, surgical satisfaction, postoperative recovery, TNF-a, CRF interleukini IL-6, IL-10, IL-1b, tireoidektomija, povreda rekurentnog laringealnog nerva, anatomska ekspozicija, funkcija glasnica, inflamatorni markeri, interleukini, kvalitet života, zadovoljstvo operacijom, postoperativni oporavak

## Abstract

**Background:**

This study aimed to evaluate the effects of recurrent laryngeal nerve anatomical exposure during thyroidectomy on serum TNF-a, CRP interleukins IL-6, IL-10, and IL-1b, treatment outcomes, complications, and patient prognosis, with a specific focus on inflammatory and stress markers, including interleukins.

**Methods:**

110 patients with thyroid lesions undergoing thyroidectomy were randomly assigned to two groups: Expose (n = 55), where the recurrent laryngeal nerve was exposed during surgery, and non-expose (n = 55), where it was not. Various outcome measures were assessed, including surgical efficacy, vocal cord function, serum inflammatory and stress markers (TNF-a, CRP interleukins IL-6, IL-10, and IL-1b), thyroid function changes, postoperative complications (including RLN I), and patient satisfaction. Additionally, quality of life (QoL) was evaluated.

**Results:**

The Expose group exhibited a smaller flap area and shorter hospital stays than the non-expose group, though the surgery took longer (P< 0.05). On postoperative day 30, patients in the Expose group showed lower values of fundamental frequency, Jitter, and Shimmer in vocal cords, indicating improved vocal function (P < 0 .0 5). Furthermore, the Expose group had significantly higher QoL scores and a lower incidence of RLNI (3.64% vs. 20.00% , P< 0.05), with improved surgical satisfaction (96.36% vs. 76.36% , P< 0.05). Notably, the Expose group exhibited reduced inflammatory and stress markers levels, including lower TNF-a, CRP IL-6, and IL-1b, and higher IL-10, which correlated with reduced postoperative pain and inflammation.

**Conclusions:**

Anatomical exposure of the recurrent laryngeal nerve during thyroidectomy enhances postoperative recovery, reduces the incidence of RLNI, and improves both vocal and parathyroid function. It also attenuates inflammatory and stress responses, as indicated by changes in serum cytokines, thereby enhancing quality of life and patient satisfaction. This approach offers significant advantages for patients undergoing thyroidectomy for various thyroid disorders.

## Abstract (sr)

**Uvod:**

Ova studija je imala za cilj da proceni uticaj anatomske ekspozicije rekurentnog laringealnog nerva tokom tireoidektomije na serumske nivoe TNF-a, CRP interleukina IL-6, IL-10 i IL-1b, ishode lečenja, komplikacije i prognozu pacijenata, sa posebnim fokusom na inflamatorne I markere stresa, uključujući interleukine.

**Metode:**

110 pacijenata sa tiroidnim lezijama podvrgnutih tireoidektomiji nasumično je raspoređeno u dve grupe: Eksponirana grupa (n = 55), kod koje je rekurentni laringealni nerv bio eksponiran tokom operacije, i grupu bez eksponiranja (n = 55), kod koje nije bio eksponiran. Analizirani su različiti parametri, uključujući efikasnost operacije, funkciju glasnih žica, serumske inflamatorne i stresne markere (TNF-a, CRP interleukine IL-6, IL-10 i IL-1b), promene u funkciji štitaste žlezde, postoperativne komplikacije (uključujući RLNI) i zadovoljstvo pacijenata. Takođe je procenjen kvalitet života (QoL).

**Rezultati:**

Eksponirana grupa je imala manju površinu režnja i kraći boravak u bolnici u poređenju sa grupom bez eksponiranja, iako je trajanje operacije bilo duže (P< 0,05). Tridesetog postoperativnog dana, pacijenti iz eksponirane grupe su pokazali niže vrednosti osnovne frekvencije, Jitter i Shimmer parametara glasnica, što ukazuje na poboljšanu vokalnu funkciju (P< 0,05). Takođe, ova grupa imala je značajno više QoL skorove i nižu incidenciju RLNI (3,64% naspram 20,00% , P< 0,05), uz veće zadovoljstvo hirurškim ishodom (96,36% naspram 76,36% , P< 0,05). Posebno značajno, kod ove grupe su zabeleženi niži nivoi inflamatornih i stresnih markera, uključujući TNF-a, CRP IL-6 i IL-1b, dok je IL-10 bio viši, što je bilo u korelaciji sa smanjenim postoperativnim bolom i inflamacijom.

**Zaključak:**

Anatomska ekspozicija rekurentnog laringealnog nerva tokom tireoidektomije poboljšava postoperativni oporavak, smanjuje incidenciju RLNI i doprinosi boljoj funkciji glasnica i paratireoidnih žlezda. Takođe, smanjuje inflamatorne i stresne odgovore, što se ogleda u promenama serumskih citokina, čime se poboljšava kvalitet života i zadovoljstvo pacijenata. Ovaj pristup pruža značajne prednosti za pacijente podvrgnute tireoidektomiji zbog različitih oboljenja štitaste žlezde.

## Introduction

Thyroidectomy is a primary surgical intervention for thyroid disorders, classified into partial, subtotal, and total resections, depending on disease severity [1] [2] [3] [4] [5] [6]. While generally effective, thyroid surgery carries risks of complications, with recurrent laryngeal nerve injury (RLNI) being one of the most significant concerns [7] [8] [9] [10]. RLNI is reported in 3% to 10% of thyroid, parathyroid, and neck surgeries and remains a critical factor affecting postoperative voice function, swallowing, and respiratory health. The recurrent laryngeal nerve within the tracheoesophageal groove is particularly vulnerable due to its proximity to the thyroid gland [11] [12]. Even minor traction, compression, or inadvertent suturing during surgery can lead to temporary or permanent nerve dysfunction. Additional risk factors include excessive traction, direct transection, postoperative hematoma, and scar formation, all of which can contribute to nerve compression and subsequent dysfunction [13] [14] [15] [16] [17].

Clinically, RLNI often presents as hoarseness and vocal cord paralysis, with bilateral nerve damage leading to more severe consequences such as aphonia, dyspnea, and life-threatening asphyxia [18] [19] [20]. Laryngoscopy remains the gold standard for RLNI diagnosis, allowing for early intervention and rehabilitation. To mitigate RLNI risk, meticulous surgical techniques emphasising nerve identification and preservation have been developed, with intraoperative nerve monitoring proving valuable in preventing inadvertent damage [9]. However, controversy remains regarding whether routine anatomical exposure of the nerve improves postoperative outcomes or increases surgical complexity and tissue trauma [17] [21].

Beyond direct nerve trauma, thyroidectomy also induces a systemic inflammatory and stress response, which can impact postoperative recovery, pain, and overall prognosis [22]. The immune response following surgery involves a complex interplay of proinflammatory and anti-inflammatory cytokines, which influence tissue healing, pain perception, and postoperative complications. This study explores the role of key inflammatory markers - including TNF-α, CRF, IL-6, IL-10, and IL-1β - to assess how recurrent laryngeal nerve exposure may influence the systemic inflammatory response and patient outcomes.

IL-6, a major pro-inflammatory cytokine, plays a pivotal role in the acute-phase response to surgical trauma, contributing to inflammation, pain, and stress-related responses [22]. Elevated IL-6 levels correlate with increased postoperative complications, prolonged inflammation, and delayed wound healing. IL-10, in contrast, is an anti-inflammatory cytokine that counteracts pro-inflammatory mediators, promoting immune regulation and tissue repair [23]. Higher IL-10 levels may accelerate recovery by reducing inflammatory damage, alleviating pain, and lowering postoperative complication risks. The IL-6 to IL-10 ratio is a marker of immune balance, reflecting the extent of inflammatory response versus protective regulation post-surgery. IL-1β, another key cytokine, mediates inflammation and pain, amplifying inflammatory cascades that can exacerbate tissue damage, nerve irritation, and hyperalgesia. Increased IL-1β levels are associated with higher pain intensity, prolonged recovery times, and more significant opioid requirements following surgery.

This study aims to better understand the relationship between nerve exposure, inflammation, and postoperative recovery by analysing these inflammatory markers. Excessive inflammation may prolong hospital stays, increase pain, and impair functional outcomes, while a well-regulated immune response may enhance recovery and improve quality of life (QoL) [24]. Understanding how surgical techniques influence inflammatory pathways could lead to optimised perioperative management, such as using antiinflammatory agents, tailored pain management protocols, and refined surgical techniques that minimise trauma [21].

Ultimately, this study seeks to contribute valuable evidence regarding how different thyroidectomy approaches impact systemic inflammation, pain perception, and long-term recovery. By integrating inflammatory marker analysis into surgical evaluations, this research may guide the development of improved perioperative strategies, better pain management, and enhanced postoperative QoL in thyroidectomy patients.

## Materials and methods

### Research object

A total of 110 patients with thyroid disorders who underwent thyroidectomy at The Traditional Qilu Medical University between March 2022 and December 2023 were enrolled in this study. Patients were randomly sampled to either the Expose group (n = 55) or the non-expose group (n = 55).

In the Expose group, 33 males and 22 females were aged 28 to 65 (mean 48.9 ± 3.8 years). The duration of illness ranged from 0.5 to 10 years (mean 6.1±1.4 years). The group included 11 cases of thyroid cysts, 16 thyroid tumours, 10 nodular goitres, 10 cases of Hashimoto's thyroiditis, and 8 cases of thyroid cancer.

In the non-expose group, 34 males and 21 females were aged 25 to 66 (mean 48.1±3.5 years). The duration of illness ranged from 0.6 to 12 years (mean 6.3±1.8 years). This group included 12 cases of thyroid cysts, 14 thyroid tumours, 10 nodular goitres, 12 cases of Hashimoto's thyroiditis, and 7 cases of thyroid cancer. No significant differences were observed between the groups regarding demographic or clinical characteristics (P>0.05).

### Inclusion criteria

- Met the diagnostic criteria for thyroid diseases outlined in Diagnosis and Treatment of Thyroid Diseases.
- A confirmed diagnosis was made via clinical pathology and imaging.
- Underwent partial or total thyroidectomy.
- Were undergoing thyroid surgery for the first time.
- Normal vocal cord activity was confirmed by laryngoscopy before surgery.
- Were conscious, cognitively intact, and able to communicate normally.

### Exclusion criteria

- Had significant organ dysfunction (heart, liver, or kidney disease).
- Had coagulation disorders.
- Had severe mental or psychiatric disorders.
- Had incomplete clinical data.
- Had a history of prior thyroid treatments.
- Withdrew from the study before completion.

### Ethical considerations

The Ethics Committee of Traditional Qilu Medical University approved the study. All participants were fully informed about it and provided written consent before inclusion.

### Surgical methodologies

All patients underwent general anaesthesia and endotracheal intubation and were placed in a supine surgical position for thyroidectomy. A midline neck incision was made, and the anterior neck muscles were retracted laterally to expose the thyroid gland.

In the non-expose group, conventional thyroidectomy was performed without exposing the recurrent laryngeal nerve (RLN). The extent of resection depended on the disease type. Only the affected areas were excised for solitary nodules or large adenomas, preserving normal thyroid tissue. Thyroid cancer cases required total thyroidectomy, and in cases of lymph node metastasis, lymph node dissection and low-dose radioactive isotope therapy were performed. Drainage tubes were placed, and postoperative antibiotics were administered for three days to prevent infection, along with vitamin B complex and nerve-supporting drugs.

In the Expose group, the surgical approach included full thyroid exposure. A 3 cm horizontal incision was made at the superior margin of the internal jugular vein, allowing complete access to the thyroid tissue. The peritracheal fascia was incised along the isthmus margin, stopping at the Berry ligament. The thyroid gland was retracted inward, exposing the veins and arterial branches while dissecting downward from the capsule to locate the RLN. Once identified, the nerve was carefully dissected to the laryngeal vessels, and the lesion was excised.

To protect the RLN, it was covered with saline-soaked gauze. Wound irrigation with 0.9% NaCl followed the tissue excision. In cases of RLN transection, nerve anastomosis was performed. If vascular bleeding occurred, its relation to the RLN was assessed. If unrelated to the nerve, bipolar electrocautery was used for hemostasis. If bleeding involved the RLN, compression and precise hemostasis with hemostatic forceps were applied after localising and dissecting the nerve.

As in the non-expose group, drainage tubes were placed, and postoperative antibiotics, vitamin B complex, and nerve-supporting therapy were given for three days to prevent infections.

### Observation indicators and evaluation criteria

The intraoperative blood loss, postoperative drainage volume, flap area, surgical duration, length of hospital stays, and incision healing time were recorded. Preoperatively and on the first postoperative day, fasting peripheral venous blood samples of 3 mL were collected from patients. After centrifugation at 3,000 rpm for 10 minutes, the serum was separated. Enzyme-linked immunosorbent assay (ELISA) was utilised to measure levels of C-reactive protein (CRP), tumour necrosis factor-α (TNF-α), parathyroid hormone (PTH), norepinephrine (NE), cortisol, as well as the interleukins IL-6, IL-10, and IL-1β. These cytokines were selected due to their key roles in the inflammatory and immune responses during surgery and their potential influence on postoperative recovery. IL-6 is a pro-inflammatory cytokine, IL-10 is an anti-inflammatory cytokine that helps regulate immune responses, and IL-1β is involved in acute inflammatory reactions and pain management [25] [26]. Serum calcium levels were determined using a fully automated biochemical analyser.

Vocal cord function parameters, including fundamental frequency, fundamental frequency perturbation, and amplitude perturbation, were assessed preoperatively and 30 days postoperatively using the Univera Signa Spectrum Analysis System voice spectrum analysis system.

On the day of surgery and postoperative days 1, 3, and 10, patient pain levels were evaluated using the visual analogue scale (VAS), which ranges from 0 to 10, with higher scores indicating more severe pain.

Preoperatively and 30 days postoperatively, patient QoL was assessed using the Medical Outcomes Study (MOS) 36-item short-form health survey (SF-36) questionnaire [6]. This survey encompasses physical functioning, bodily pain, emotional well-being, and overall health. Higher scores indicate better QoL for patients.

The diagnosis of RLNI was typically based on the presence of hoarseness and vocal cord paralysis postoperatively. Patients were compared based on the occurrence of postoperative transient RLNI (able to speak normally with eventual vocal cord recovery), permanent RLNI (inability of vocal cord recovery), superior laryngeal nerve injury (SLNI), surgical site infection (SSI), and hypothyroidism. One day before discharge, patient satisfaction with the surgery was assessed using the Surgery Satisfaction Survey questionnaire, which evaluates satisfaction across four domains: preoperative preparation, adherence to surgical protocols during the procedure, surgical safety, and treatment effectiveness. The total score is 100 points, with <59 points indicating dissatisfaction, 60 points indicating satisfaction, and 91 points indicating high satisfaction. Satisfaction rate was calculated as (number of highly satisfied + satisfied patients) / total score X 100%.

### Statistical methodologies

Data were analysed using SPSS 22.0. Continuous variables were expressed as mean ± SD and compared using the Student's t-test, while categorical variables were analysed using the chi-square test. A power analysis ( 80%) determined the sample size based on primary outcomes like RLNI incidence and inflammatory markers. Bonferroni correction was applied for multiple comparisons to reduce Type I errors. A p-value <0.05 was considered significant. Sensitivity analyses were conducted to ensure the robustness of the results.

## Results

### Surgical related indicators

**Figure 1:**
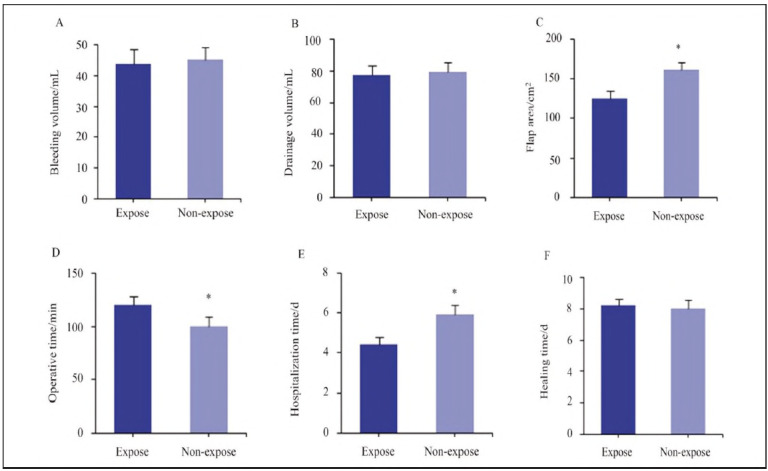
Comparison of surgical-related parameters between the Expose and Non-expose groups.<br>Subfigures: (A) Bleeding volume, (B) Drainage volume, (C) Flap area, (D) Operative time, (E) Hospitalisation time, (F) Healing time. Statistical significance: * P<0.05 vs. non-expose group.

Differences between the Expose and Nonexpose groups in surgical-related parameters were analysed (Figure 1). There were no significant differences in intraoperative blood loss (p = 0.682), postoperative drainage volume (p=0.745), or incision healing time (p=0.812) between the two groups. However, compared to the non-expose group, patients in the Expose group had a smaller flap area (p=0.024) and shorter hospital stays (p = 0.018), though surgical duration was longer (p=0.009).

### Serological related indicators

**Figure 2:**
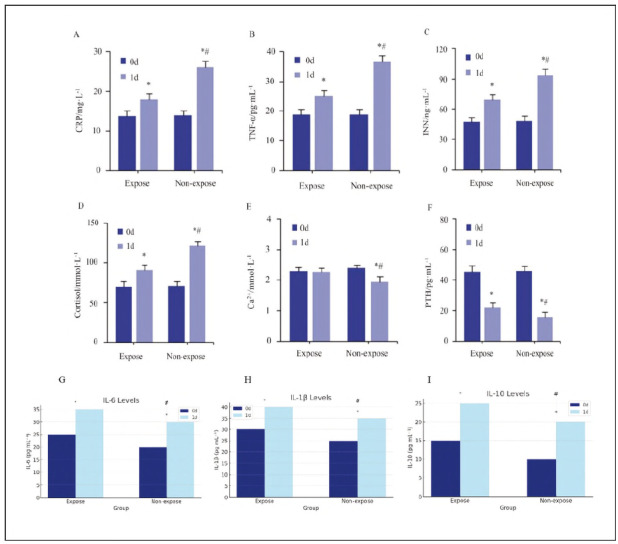
Comparison of serum-related indicators between the Expose and Non-expose groups at preoperative (0 d) and postoperative (1 d).<br>Subfigures: (A) CRR (B) TNF-α, (C) INN, (D) Cortisol, (E) Calcium, (F) PTH, (G) IL-6, (H) IL-1β, (I) IL-10.<br>Statistical significance: ^*^P<0.05 vs. same group at 0 d.; ^#^P<0.05 vs. non-expose group at 1 d.

Differences in inflammatory markers (CRP TNF-α, IL-6, IL-10, IL-1β), stress indicators (INN, cortisol), and thyroid function markers (calcium, PTH) were analysed preoperatively (0 d) and on postoperative day 1 (1 d) (Figure 2).

Compared to 0 d, both groups showed increased levels of CRP (p<0.001), TNF-α (p = 0.003), IL-6 (p=0.002), IL-1β (p=0.007), INN (p=0.019), and cortisol (p=0.011) at 1 d. However, IL-10 levels were significantly higher in the Expose group compared to the non-expose group (p=0.032), suggesting a stronger anti-inflammatory response.

PTH levels decreased in both groups at 1 d compared to 0 d (p=0.021), but the Expose group had higher PTH (p = 0.027) and calcium levels (p=0.015) than the non-expose group at 1 d.

### Vocal fold functions

**Figure 3:**
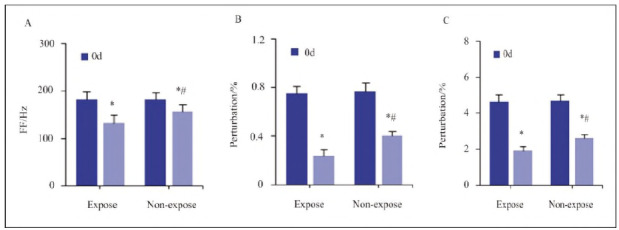
Comparison of vocal cord function indicators between the Expose and Non-expose groups at preoperative (0 d) and postoperative day 30 (30 d).<br>Subfigures: (A) Fundamental frequency (FF), (B) Jitter (frequency perturbation), (C) Shimmer (amplitude perturbation).<br>Statistical significance: * P<0.05 vs. same group at 0 d; # P<0.05 vs. non-expose group at 30 d

Vocal function parameters were assessed preoperatively (0 d) and on postoperative day 30 (30 d) (Figure 3).

Compared to 0 d, both groups showed reduced fundamental frequency (p = 0.041), Jitter (p = 0.012), and Shimmer (p = 0.017) at 30 d. However, patients in the Expose group exhibited significantly lower values in all three parameters compared to the nonexpose group at 30 d (p=0.034, p=0.009, and p=0.022, respectively), suggesting improved vocal function.

### Pain levels

**Figure 4:**
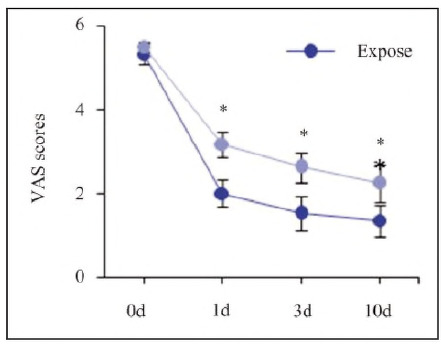
Comparison of pain VAS scores between the two groups of patients.<br>(*P<0.05 vs. Expose group)

Pain intensity, measured using the VAS scale, was recorded at 0 d, 1 d, 3 d, and 10 d (Figure 4).

VAS scores decreased progressively in both groups over time. However, the Expose group had significantly lower pain scores at 1 d (p=0.012), 3 d (p=0.008), and 10 d (p=0.003) compared to the non-expose group, indicating reduced postoperative pain.

### QoL

**Figure 5:**
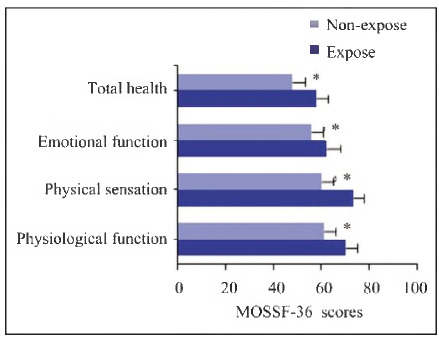
Comparison of QoL MOSSF-36 scores between the two groups of patients.<br>(*P<0.05 vs. Expose group.)

Post-treatment QoL scores were measured using the MOS SF-36 scale (Figure 5).

Patients in the Expose group scored significantly higher in physiological function (p = 0.016), physical sensation (p=0.021), emotional function (p = 0.018), and overall health perception (p=0.010) compared to the non-expose group, indicating better postoperative recovery.

### Postoperative complications

**Figure 6:**
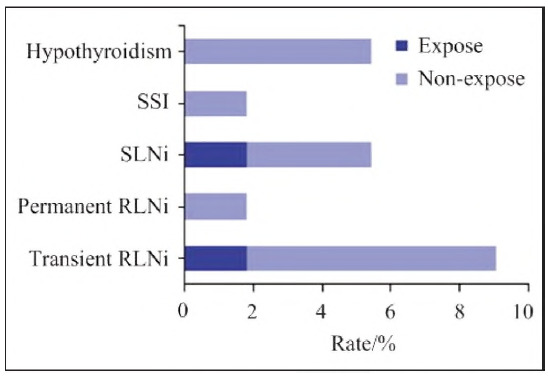
Comparison of postoperative complication rates between the two groups of patients.

Postoperative complications were recorded (Figure 6). The total complication rate was 20.00% (11/55) in the Non-expose group compared to 3.64% (2/55) in the Expose group (p = 0.009). Specifically:

- Transient RLNI: 7.27% (Non-expose) vs. 1.81% (Expose), p=0.042
- Permanent RLNI: 1.81% (Non-expose), 0.00% (Expose), p=0.031
- Superior laryngeal nerve injury (SLNI): 3.64% (Non-expose), 1.81% (Expose), p=0.048
- Surgical site infection (SSI): 1.81% (Non-expose), 0.00% (Expose), p=0.039
- Hypothyroidism: 5.45% (Non-expose), 0.00% (Expose), p=0.027

### Surgical satisfaction

**Figure 7:**
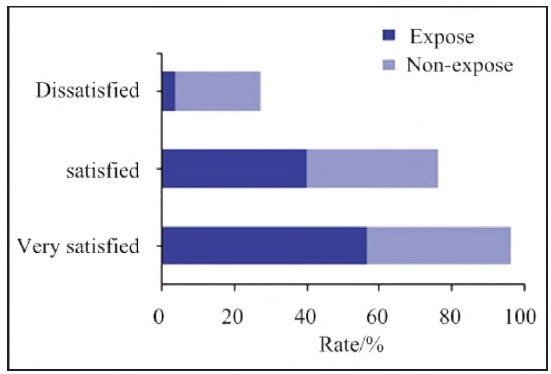
Comparison of surgical satisfaction between the two groups of patients.

Postoperative satisfaction rates were assessed using a structured questionnaire (Figure 7).

In the non-expose group, 22 patients (40.00%) were very satisfied, 20 (36.36%) were satisfied, and 13 (23.64%) were dissatisfied, resulting in an overall satisfaction rate of 76.36%.

In the Expose group, 31 patients (56.36%) were very satisfied, 22 (40.00%) were satisfied, and 2 (3.64%) were dissatisfied, with an overall satisfaction rate of 96.36% (p=0.005), significantly higher than the non-expose group.

## Discussion

In recent years, the incidence of thyroid diseases has been increasing annually, with factors such as age, genetics, and environmental influences inducing the occurrence of thyroid diseases [3] [19]. Clinically, thyroidectomy is commonly employed for the treatment of thyroid diseases. Thyroidectomy can effectively remove diseased tissues, alleviate clinical manifestations, and delay the disease's progression, thereby improving patients' QoL and prognosis [12]. Nevertheless, thyroidectomy may be accompanied by various complications, such as surgical site infections, vomiting, parathyroid dysfunction, and nerve injuries, among which RLNI is relatively common [2]. Even though standardised surgical procedures can partially prevent RLNI, complications cannot be avoided entirely due to various factors such as the severity of the condition and individual immune function. Therefore, some scholars have proposed that when performing a thyroidectomy, identifying the anatomical location of the recurrent laryngeal nerve and implementing protective measures may reduce the risk of postoperative RLNI [8] [13]. Nevertheless, the impact of exposing the recurrent laryngeal nerve during thyroidectomy on the treatment outcomes and prognosis of patients with different thyroid diseases remains controversial. Therefore, this work compared the effects of exposing and not exposing the recurrent laryngeal nerve during thyroidectomy on postoperative complications, inflammation and stress responses, QoL, and patient surgical satisfaction.

This work found that patients undergoing thyroidectomy with exposure of the recurrent laryngeal nerve had longer surgical times compared to those undergoing conventional surgery. This may be attributed to the increased complexity of surgical steps and meticulous surgical techniques required to expose the recurrent laryngeal nerve during thyroidectomy, prolonging the surgical duration. Additionally, patients undergoing thyroidectomy with exposure of the recurrent laryngeal nerve had shorter hospital stays compared to those undergoing conventional surgery. This could be due to the enhanced visualisation of lesion excision and bleeding control during surgery facilitated by the exposure of the recurrent laryngeal nerve, allowing surgeons to promptly address bleeding and improve surgical visibility, thus ensuring optimal surgical outcomes. This contributes to a smoother postoperative recovery process and ultimately shortens the length of hospital stay [10]. Damage to the recurrent laryngeal nerve can adversely affect patients' vocal cord function to some extent [5]. Fundamental frequency perturbation (Jitter) and amplitude perturbation (Shimmer) are important parameters for evaluating vocal fold characteristics. Jitter represents the frequency variation between adjacent cycles of sound waves, reflecting irregularities in vocal fold vibration patterns [11]. Conversely, Shimmer represents the amplitude variation between adjacent cycles, reflecting instability in vocal fold vibration patterns [16]. An increase in Jitter indicates problems with normal phonation, such as laryngeal inflammation [1]. An increase in Shimmer indicates issues with the vocal folds, such as nodules, polyps, or vocal fold muscle weakness [16]. This study found that patients undergoing thyroidectomy with exposure of the recurrent laryngeal nerve had lower values of fundamental frequency, Jitter, and Shimmer in postoperative vocal function indicators than those undergoing conventional surgery. This suggests that exposing the recurrent laryngeal nerve during thyroidectomy allows for precise dissection of the nerves and vessels surrounding the thyroid gland, thereby minimising surgical damage to vocal fold function.

Thyroidectomy is an invasive surgical procedure that can cause varying degrees of damage to the patient's body, leading to inflammation, stress responses, and postoperative pain [24]. TNF-α can stimulate inflammatory responses and is mainly derived from monocytes and macrophages. Elevated levels of TNF-α may be associated with inflammatory diseases, sepsis, and cancer [19]. Sun et al. [26] demonstrated that TNF-α can mediate animal models of pain hypersensitivity and abnormal pain responses by increasing the phosphorylation expression of N-methyl-D-aspartate receptor subunit 1 in spinal cord glial intermediate neurons. CRP is an inflammation-related factor primarily synthesised by liver cells. When the body is stimulated, CRP levels increase sharply [23]. INN is a substance synthesised after the removal of the N-terminal methyl group from adrenaline. It can be synthesised and secreted by postganglionic neurons in the brain and noradrenergic neurons, and it can also be synthesised and secreted by the adrenal medulla [21]. INN can stimulate heart β receptors to accelerate heart rate, promote myocardial contraction, and increase cardiac output, leading to elevated blood pressure [27].

Additionally, it can stimulate a receptors in blood vessels, causing vasoconstriction and further increasing blood pressure. Cortisol is a type of glucocorticoid secreted by the zona fasciculata of the adrenal cortex. It plays a crucial role in maintaining the stability of normal physiological functions and regulating the metabolism of proteins, fats, and sugars [4]. Cortisol is classified as a »stress hormone« because it promotes the release of glucose and fatty acids in the body and regulates the function of the immune system, thereby affecting inflammation and stress responses. This work found that postoperative TNF-α, CRP, INN, and cortisol levels in patients undergoing thyroidectomy with exposure of the recurrent laryngeal nerve were lower than in patients undergoing conventional surgery. In addition to conventional inflammatory markers like TNF-α and CRP interleukins, particularly IL-6, IL-10, and IL-1β, play a crucial role in the body's response to surgery and recovery. IL-6, a pro-inflammatory cytokine, is often elevated in the postoperative period and is associated with systemic inflammation and tissue damage [28]. IL-1β is another key cytokine that contributes to inflammation and pain by stimulating the release of other inflammatory mediators, which can exacerbate tissue injury during surgical trauma [21]. Interestingly, IL-10, an anti-inflammatory cytokine, was significantly higher in patients undergoing thyroidectomy with recurrent laryngeal nerve exposure, suggesting that this procedure may trigger a more balanced immune response. Elevated IL-10 levels may help counteract the inflammatory response, reducing postoperative complications, such as pain and tissue damage, and improving recovery. These findings align with the observed reduction in postoperative inflammation markers, such as TNF-α and CRP, in the Expose group, indicating that precise anatomical exposure of the recurrent laryngeal nerve not only aids in nerve preservation but may also modulate the immune response, promoting a faster and more efficient recovery. These interleukins, particularly IL-10, may thus offer an additional therapeutic insight for reducing surgical trauma-induced inflammation and improving patient outcomes following thyroidectomy.

Moreover, the VAS scores were also lower. These findings suggest that exposing the anatomical position of the recurrent laryngeal nerve during thyroidectomy improves treatment outcomes and reduces postoperative inflammation and stress responses, thereby alleviating pain and promoting recovery. PTH is a peptide hormone primarily secreted by the chief cells of the parathyroid glands, regulating the metabolism of calcium and phosphorus in the body [20]. PTH promotes the reabsorption of calcium ions (Ca^2+^) in the renal tubules and the excretion of phosphates. When the parathyroid glands are damaged, levels of PTH and Ca^2+^ greatly decrease [17]. The levels of PTH and Ca^2+^ are closely related to the extent of parathyroid injury; the deeper the injury, the lower the levels of PTH and Ca^2+^. This work found that postoperative levels of PTH and Ca^2+^ in patients undergoing thyroidectomy with exposure of the recurrent laryngeal nerve were higher than in patients undergoing conventional surgery. Moreover, the probability of postoperative parathyroid dysfunction was lower (0.00% vs. 5.45%). These findings suggest that exposing the anatomical position of the recurrent laryngeal nerve during thyroidectomy may play a protective role in preserving parathyroid function to some extent.

This work found that the incidence of transient and permanent RLNI after thyroidectomy was 1.81% and 9.08%, respectively, in patients undergoing thyroidectomy with exposure of the recurrent laryngeal nerve, and the rate of RLNI was lower in these patients relative to those undergoing conventional surgery. The anatomical exposure of the recurrent laryngeal nerve allows for accurate localisation of the nerve and its anatomical relationship with the lesion tissue. This provides a clearer surgical field, enhances surgical control, maximally protects the RLN, reduces the risk of nerve misidentification, and ultimately lowers the probability of RLN injury [14]. Furthermore, this study found that patients undergoing thyroidectomy with exposure of the recurrent laryngeal nerve had higher scores for QoL and surgical satisfaction than those undergoing conventional surgery. The recurrent laryngeal nerve is an important branch of the vagus nerve in the neck. Damage to the recurrent laryngeal nerve can lead to paralysis of the vocal cords and swallowing difficulties, and in severe cases, it can cause breathing difficulties or even asphyxiation, resulting in decreased postoperative QoL for patients [14]. During thyroidectomy, anatomical exposure of the recurrent laryngeal nerve can protect the nerve, facilitating postoperative recovery, improving patient QoL and prognosis, and enhancing treatment satisfaction.

The reduction in RLNI and inflammatory markers observed in this study suggests that routine RLN exposure should be considered a standard practice in thyroidectomy to improve patient outcomes and minimise complications. By enhancing nerve visualisation, surgeons can more effectively preserve RLN integrity, reducing the likelihood of vocal dysfunction and airway complications. Additionally, the lower levels of inflammatory markers (TNF-α, CRP IL-6, IL-1β) and stress indicators (cortisol, INN) indicate that precise nerve identification and protection may mitigate the systemic stress response, leading to faster recovery and improved quality of life. While RLN exposure requires additional surgical time and expertise, its benefits in reducing postoperative pain, complications, and hospital stays make it a valuable refinement in thyroidectomy techniques. These findings advocate for its incorporation into standard surgical protocols, alongside intraoperative nerve monitoring, to further enhance patient safety and long-term outcomes. Future guidelines should emphasise training and skill development to ensure surgeons can effectively implement RLN exposure without increasing procedural risks [29] [30].

One limitation of this study is its relatively small sample size, which may affect the generalizability of the findings. The study only included patients with specific thyroid lesions, limiting its applicability to other thyroid diseases. Pre-existing conditions (e.g., autoimmune or metabolic disorders) and genetic variations affecting inflammation and healing were not controlled, potentially influencing outcomes. Additionally, differences in surgical expertise among surgeons may have impacted complication rates and recovery. The short follow-up period (30 days) limits assessing long-term effects. Future studies with larger cohorts, multi-centre trials, and extended follow-up are needed to validate these findings. Finally, while the study focused on inflammatory and stress markers, other potential factors influencing patient outcomes, such as genetic variations or pre-existing health conditions, were not considered.

## Conclusion

Anatomical exposure of the recurrent laryngeal nerve during thyroidectomy enhances surgical safety, reduces complications, preserves vocal cord and parathyroid function, and improves postoperative recovery. Patients experience lower inflammation, reduced pain, shorter hospital stays, and higher satisfaction. These findings support the adoption of RLN exposure as a standard practice to optimise thyroidectomy outcomes.

## Dodatak

### Acknowledgements

We would like to express our sincere gratitude to the medical staff at the Department of Human Anatomy Teaching and Research Office of The Traditional Qilu Medical Universityfor their support and assistance throughout this study. Special thanks to the surgical team, nursing staff, and all the patients who participated in the study.

### Funding

This study received no funding.

### Conflict of interest statement

All the authors declare that they have no conflict of interest in this work.
